# Clinical Reports—New Orleans

**Published:** 1893-06

**Authors:** Robert Morris

**Affiliations:** Resident Student and Ambulance Surgeon of N. O. Charity Hospital


### CLINICAL REPORTS—NEW ORLEANS.

[By Robert Morris, Resident Student and Ambulance Surgeon of N. O. Charity Hospital.]

### A YEAR ON THE AMBULANCE.

A few preliminary words before enumerating the cases and the treatment pursued, may not be amiss.

This compilation includes those cases which came under my care on the ambulance and in the amphitheatre, and as I attended to only my pro rata of the ambulance calls and the amphitheatre cases, it only represents a small portion of the surgical work of the hospital.

The amphitheatre cases are those emergency cases which are brought to the hospital and demand immediate treatment.

Each intern is on duty certain days, and when such cases are brought in they are called, and if the case is of much gravity, the House Surgeon or Assistant House Surgeon is called in to advise or operate.

The after-treatment of many of the cases cannot be given, as they were sent to wards over which I had no jurisdiction, but the result will be mentioned as accurately as possible.

For convenience, I will divide the cases into surgical, medical, and toxicological.

From April 1, 1892, to April 1, 1893, I attended to 271 ambulance calls, and 205 amphitheatre cases. In 15 of the calls the patient was dead on arrival; so these will not be considered.

### SURGICAL.

*Amputations.*—There were two amputations of the thigh, eight of the leg, sixteen of the arm, four of the foot, and ten of the fingers and toes.

The flaps were made by the circular method in the thigh and arm, the modified circular in the leg, the posterior flap in the foot, and the pocket incision in the fingers and toes, when the injury of the part would permit.

Both of the thigh amputations died; in one case the amputation was due to spreading gangrene following a compound fracture, the other was due to gangrene resulting from an embolism of the femoral artery,—unfavorable cases.

In operating, all aseptic and antiseptic precautions are carefully observed; the field of operation is thoroughly cleansed with soap, alcohol, carbolized water, and corrosive sublimate solution, 1-3000, while sterilized and carbolized towels are placed around the operative area. The instruments are sterilized in pure water, to which has been added a small quantity of bicarbonate of sodium, and placed in a carbolized solution, 2 to 5%. The operator's hands are cleansed in a similar manner to the field of operation.

The wound is dressed with iodoform and bichloride gauze; this dressing remains for six or seven days, depending upon the temperature and the condition of the dressing. The drainage tube is removed at the first dressing.

*Fractures.*—There were seven fractures of the skull, four of which were trephined. Of the trephined cases, three recovered, while all of the non-trephination cases recovered.

In trephining, the usual antiseptic precautions are observed in cleansing the scalp and the instruments, but when the button is removed, sterilized water alone is used, or a mildly carbolized solution, so as to avoid all danger of exciting a cerebritis.

Aspiration is the usual manner of ascertaining the presence of a sub-dural hemorrhage, and when discovered a slit is made in the dura, and as much blood as can be consistently removed, is withdrawn. The dural opening, if the hemorrhage is extensive, is drained with a small rubber tube, around which is placed sterilized gauze. The wounds are dressed from time to time, depending upon the severity of the scalp injury, and the amount of subdural hemorrhage.

One case brought in on the ambulance was particularly interesting, as it was of unusual severity, and presented very pronounced symptoms of irritation of motor area. The patient, a negro 22 years of age, was knocked down by a street car while attempting to jump aboard. An examination revealed the following:

A small wound of temporal region and an extensively contused wound of scalp over the left parietal bone, under which was a fissured fracture about two inches in length, extending backward from a point on the coronal suture about one and a half inches from its juncture with the sagittal. In addition to this fracture, there was a separation of the left parietal and frontal bones. The amount of separation was about one-eighth of an inch, the separation extending from the anterior end of the spheno-parietal suture to the junction of the sagittal with the coronal.

While the patient was on the table, he had two of the most typical convulsions, from pressure on the motor area, that I ever witnessed; first there was a spasmodic contraction of the frontal muscle, then, after an interval of a few seconds the right arm became clonically convulsed, which passed to the right leg, then became general. The seizures lasted for a short time only, after which patient would again become somnolent.

A button of bone was removed very close to the line of fracture, by the Assistant House Surgeon, Dr. Bloom, an inspirating needle was passed through the dura-mater, and a small quantity of blood was withdrawn. The wounds were dressed so as to give free exit to any blood that might escape through this minute opening in the dura.

The patient recovered. The after-treatment was supervised by Dr. Souchon, to whose ward he was assigned.

*Nasal Bones.*—There were only two cases of fracture of the nasal bones, and these were treated with strips of rubber adhesive plaster, the bones being held in position by waddings of gauze placed on the outer side of the bones and held in that manner by the strips of plaster. The result in each case was very satisfactory,—only slight deformity.

*Fracture of Inferior Maxilla.*—Two cases were treated, one by a pasteboard splint supported by a Barton's bandage, the other by wiring the adjoining teeth of the two fragments. The result was satisfactory.

*Fracture of Clavicle.*—There were six cases. These were treated with a Valpeau's bandage. There was some deformity in all the cases.

*Humerus.*—There was one fracture of the anatomical neck, one of the surgical, three T fractures of the inferior extremity, and one complete separation of lower epiphysis. The two former cases were treated by bandaging the arm to the chest with liquid glass dressing, strengthened by a pasteboard shoulder cap; the latter were splinted with an obtuse angular, tin splint. This dressing remained twelve or fourteen days, when passive motion was instituted and practiced every third or fourth day, if it did not produce severe pain or inflammation.

*Forearm.*—There were twenty-one cases of fracture of the bones of the forearm. In six of these cases both bones were broken, two of which were compound. The radius was fractured twelve times, eight of which were Colle's, the remaining were of the shaft; one of these was open. The ulna was broken three times; one of the olecranon process, the others of the shaft.

The cases were treated by splinting the forearm midway between pronation and supination, if the break was below the radial attachment of the pronator radii teres, if above, in complete supination. The fractures of the radius at the lower third, were splinted in a similar manner, observing that the palmar splint did not project beyond a line corresponding to the superficial palmar arch, and that the palmar splint was so padded that the radial convexity was preserved. This manner of applying the splints tends to retain the fragments in their proper position, and allows free movements of the fingers, so that no tendinous adhesions could form. One case was treated by Moore's method, that is, bands of rubber adhesive plaster wound snugly around the wrist, with very satisfactory results.

One of the cases of Colle's fracture was not pleasing in its result. The fingers were stiff, the bone somewhat deformed, and the wrist movements very much retarded five months after the receipt of the injury.

The fractures of the shaft of the ulna were splintered midway between the pronation and supination. Fracture of the olecranon was treated by completely extending the forearn, and maintaining it in that position.

The cutaneous wounds of the open fracture were carefully and thoroughly cleansed, and irrigated with bichloride solution, 1-2000, before dressing, and redressed as often as the case demanded.

*Fingers.*—Nine fractures of the fingers were treated, all with a palmar splint, utilizing the sound fingers as lateral supports.

*Ribs.*—There were five cases of fracture of the ribs. These were invariably treated with rubber adhesive plaster, either in strips or in single width of five inches. The plaster started one or two inches beyond the vertebra, on the sound side, and extended the same distance beyond the sternum in front, immobilizing the fracture.

*Fracture of the Ilium.*—Only two fractures of the ilium, and these were of the crest. In both cases wide strips of adhesive plaster were placed around the body, completely encircling the pelvis.

One of the cases left the hospital early, and the result could not be ascertained, but the other remained five weeks, and was discharged with complete osseous union of the two fragments.

